# Equity in the Pandemic Treaty: Access and Benefit-Sharing as a Policy Device or a Rhetorical Device?

**DOI:** 10.1017/jme.2023.59

**Published:** 2023

**Authors:** Abbie-Rose Hampton, Mark Eccleston-Turner, Michelle Rourke, Stephanie Switzer

**Affiliations:** 1: DEPARTMENT OF GLOBAL HEALTH & SOCIAL MEDICINE, KING’S COLLEGE LONDON, UNITED KINGDOM; 2: LAW FUTURES CENTRE, GRIFFITH UNIVERSITY, AUSTRALIA; 3: STRATHCLYDE LAW SCHOOL, UNIVERSITY OF STRATHCLYDE, SCOTLAND

**Keywords:** Pandemic Treaty, Access and Benefit-Sharing, Pandemic Influenza Preparedness Framework, Pathogen Sharing, Access to Medicines

## Abstract

Equity is a foundational concept for the new World Health Organization (WHO) Pandemic Treaty. WHO Member States are currently negotiating to turn this undefined concept into tangible outcomes by borrowing a policy mechanism from international environmental law: “access and benefit-sharing” (ABS).

World Health Organization (WHO) Member States are currently negotiating a “convention, agreement or other international instrument for pandemic preparedness and response” — the so-called “Pandemic Treaty.”^1^ This Treaty is intended to address global policy failures in outbreak prevention, preparedness, and response during the COVID-19 pandemic. Central to both the justification provided by proponents of the Treaty, and the negotiation process thus far, is the issue of equity. Proponents argue that the Treaty should be grounded in “norms of solidarity, fairness, transparency, inclusiveness and equity” to overcome the shortcomings of the international COVID-19 response.^2^

Equity has been an ever-present feature of negotiations, and has moved from being a vague ambition for the Treaty to something that should be “a principle, an indicator and an outcome of pandemic prevention, preparedness and response.”^3^ Much of the discussion around equity has centered on “timely access to affordable, safe and efficacious pandemic response products, among and within countries, including between groups of people irrespective of their social or economic status.” Negotiators are looking to operationalize the concept of equity by “establishing a comprehensive system for access and benefit-sharing” that would include “measures to ensure equitable and affordable access to quality, safe and effective pandemic response products, including those drawn from strategic stockpiles, and their equitable distribution.”

The Pandemic Treaty thus looks to an access and benefit-sharing (ABS) transaction as a way to operationalize equity in global health law. Article 10 of the February 2023 zero draft of the Treaty stipulates that the “WHO Pathogen Access and Benefit-Sharing System (the “PABS System”)” is to be established – connecting the “rapid, systematic and timely” sharing of “pathogens with pandemic potential” and “the genomic sequence of such pathogens” (access) with the sharing of the “benefits arising from facilitating access to pathogens with pandemic potential” in a manner which is understood to be fair and equitable (benefit-sharing).^4^ This promotion of ABS within the Pandemic Treaty is concerning given that the ABS mechanism has long proven incapable of delivering equitable outcomes under international law.

## Origins of ABS in International Environmental Law

ABS seeks to ensure access to genetic resources and sharing of benefits associated with their use. It is a policy mechanism that originated from the United Nations (UN) 1992 Convention on Biological Diversity (CBD). The CBD reaffirmed that States have sovereign rights over their genetic resources, and the authority to determine the rules about accessing these resources. Thus, the CBD expects potential users of sovereign genetic resources to seek prior informed consent from the originating State before using those resources in research and development (R&D), and for the provider State and the user party to come to a mutual agreement about how to share the benefits of R&D — in a fair and equitable way. This “decidedly transactional approach relied on bilateral contracts” to implement the CBD’s ABS provisions,^5^ with the hope that the ABS transaction would generate market incentives to conserve biodiversity and sustainably use genetic resources.^6^

The mechanism was developed further in 2010, when the Conference of the Parties to the CBD adopted the Nagoya Protocol on Access to Genetic Resources and the Fair and Equitable Sharing of Benefits Arising from their Utilization (“Nagoya Protocol”), which clarified the CBD’s provisions on ABS transactions. The Nagoya Protocol clarified that Parties to the CBD were to create fair and non-arbitrary procedures for accessing genetic resources. Acknowledging that the bilateral contractual model of ABS introduced by the CBD was often too simplistic to work in the real world, the Nagoya Protocol further encouraged Parties to cooperate when genetic resources were found in more than one sovereign territory, pushing Parties to consider a global multilateral benefit-sharing mechanism for these sorts of transboundary situations. It also made provision for the recognition of specialized international ABS instruments for specific types of genetic resources, under which the provisions of the Nagoya Protocol would not apply. ABS was a policy mechanism originally designed to contribute to the conservation of biological diversity and the sustainable use of its components, but despite its limitations,^7^ it was later transposed into public health with the adoption of WHO’s Pandemic Influenza Preparedness Framework (PIP Framework).

## PIP Framework: ABS in Public Health

The 2011 PIP Framework is the only international pathogen-specific multilateral ABS mechanism to date. It explicitly frames access to pathogen samples — specifically, influenza virus of human pandemic potential — in exchange for medical countermeasures. It recognizes the “sovereign rights of States over their biological resources” and seeks to create a “fair, transparent, equitable, efficient, effective system,” placing “access to vaccines and sharing of other benefits” on equal footing with the sharing of influenza virus samples. Under the PIP Framework, WHO Member States can share their sovereign pandemic influenza virus samples with the Global Influenza Surveillance and Response System (GISRS), the global network of WHO-affiliated influenza laboratories. WHO is then able to share these samples with third-party entities, such as pharmaceutical companies and vaccine manufacturers. In exchange, these third-party companies are required to share associated benefits, such as vaccines and therapeutics, with the WHO. Through the Framework’s use of binding Standard Material Transfer Agreements (SMTAs), vaccine manufacturers are typically expected to commit “at least 10% of real time pandemic vaccine production to WHO” or to “[r]eserve at least 10% of real time pandemic vaccine production at affordable prices to WHO.”^8^ These commitments are expected to contribute to the production of a virtual vaccine stockpile, which would materialize in the event of an influenza pandemic for onward distribution to States “according to public health risk and need.”^9^

The PIP Framework has been celebrated as “milestone in global governance for health,”^10^ with SMTAs seen as its “greatest accomplishment for equity,”^11^ but such celebrations are premature — as the Framework remains untested. It is unclear whether the expectations surrounding its ability to procure and distribute benefits in an equitable manner will align with reality during an influenza pandemic. There are concerns around the limited bargaining power of WHO as the mediator of the ABS agreements under the PIP Framework as well as insufficient benefit-sharing commitments. There is also the risk that the Framework, given its reliance on a virtual stockpile, will ultimately be stymied by unfulfilled promises, rampant vaccine nationalism, and export restrictions. Together with the failure of the Framework to include little more than a vague overview of how benefits should be distributed (if, indeed, they can be procured), there remain doubts about the delivery of promised benefits under the PIP Framework during a future influenza pandemic.^12^

## Calls for ABS in the Pandemic Treaty

Drawing from the PIP Framework, ABS has been suggested as a potential mechanism to achieve equity in the Pandemic Treaty. It is thought that ABS will solve two problems: (1) ensuring scientists and public health researchers have access to pathogen samples and associated genetic sequence data and (2) securing products like diagnostic kits, vaccine doses, and therapeutics for WHO to distribute to countries in need during a pandemic. While both issues presented themselves during the COVID-19 pandemic, they were not explicitly connected through the ABS mechanism — instead addressed as standalone issues.

Throughout the COVID-19 pandemic, scientists from around the world have isolated the SARS-CoV-2 virus from patients’ nasal swabs, and the genetic code of these virus isolates have been sequenced and uploaded to online sequence repositories for other researchers and public health practitioners to monitor and analyze. As viruses evolve as they spread, genetic sequencing has become a vital part of the public health response, allowing researchers to assess the spread of SARS-CoV-2, monitor the emergence of new variants, and generate diagnostics, therapeutics, and even vaccines. Yet, beyond genetic sequences, scientists still require access to physical samples of pathogens for most R&D, and in the early days of COVID-19, China was not forthcoming with physical samples of the virus, limiting the international response. Further, where countries shared information about SARS-CoV-2, as seen when South Africa promptly reported Omicron as a variant of concern, this was met with trade and travel sanctions rather than solidarity and support.^13^ Such nationalist actions are not new in public health, but they clearly disincentivize countries from sharing public health information.

The second problem is access to medical countermeasures (particularly vaccines) for lower and middle-income countries (LMICs). COVAX was created during COVID-19 to facilitate vaccine equity, acting as the key purchasing agent for the world, pooling demand, and exercising significant market shaping and equitable allocation powers. However, as COVAX failed by some margin to meet its distribution goals,^14^ countries like South Africa and India railed against treating intellectual property (IP) rights as “sacrosanct”^15^ — seeking with limited success to relax the application of the World Trade Organization (WTO) Agreement on the Trade Related Aspects of Intellectual Property (TRIPS Agreement). There remain systemic barriers to LMICs in manufacturing their own medical countermeasures during a pandemic, and without significant changes to these systemic barriers, they will remain reliant on pooling and distribution mechanisms facilitated by the WHO and other international organizations.

## The Pandemic Treaty: Can ABS Solve the Problems it is Designed to Solve?

The COVID-19 response has seen major issues with both access to pathogenic genetic resources (SARS-CoV-2 samples) and the ability of LMICs to access vaccines and medical countermeasures, and in overcoming these obstacles in future pandemics, States are seeking to connect these problems by creating an ABS mechanism under the Pandemic Treaty. However, the ability of the PIP Framework to deliver benefits has not been tested, and there are several reasons to believe it will fail to deliver promised benefits during a future pandemic. Beyond the specific concerns with the PIP Framework, there are also broader concerns with the use of the ABS mechanism in the public health space.

ABS reaffirms countries’ sovereign rights over their genetic resources, which they could trade for a fairer share of the benefits of scientific research and development; however, far from decolonizing science and repositioning the Global South to take advantage of public health R&D conducted in the Global North, ABS has become “a legal compliance mechanism to justify a ‘business as usual’ approach without fundamentally shifting power relations or economic disparities.”^16^ Many ABS agreements remain confidential, making it impossible to assess whether the outcomes are, in fact, equitable. Additionally, the application of ABS rules in the global health policy arena has already led to fundamentally anti-scientific outcomes where, for example, vaccines have been developed using suboptimal pathogen strains due to researchers being unable to negotiate access to the most appropriate samples.^17^ As countries do not cede their sovereign rights over their pathogenic genetic resources, multilateral ABS mechanisms like the PIP Framework are always vulnerable to being undermined by bilateral ABS deals.^18^ The major concern is that ABS incentivizes countries to withhold samples from the international scientific community in the hopes that they can secure more favorable benefit-sharing terms through bilateral ABS negotiations.

ABS has proven incapable of delivering equitable outcomes in international environmental law for the last three decades. If Member States genuinely want to achieve anything approaching equitable outcomes in the next pandemic, they must be look to regional capacity building, technology, and know-how transfer before the next pandemic.

If ABS is negatively impacting science and not delivering equity, it remains unclear why it has endured as a potential solution under global health law. Nor is it clear why the PIP Framework has become the model for the development of an ABS system for other pandemic pathogens under the Pandemic Treaty.^19^ Drawing from international biodiversity law and then the PIP Framework, the establishment of these structures has provoked a form of path dependency, whereby ABS is equated with equity, making it “progressively more difficult to return to the initial point when multiple alternatives were still available.”^20^ If WHO Member States were to design solutions from scratch to address the problems of access to pathogen samples and access to vaccines and other medical countermeasures, these issues would be dealt with separately, rather than connected through the ABS mechanism; yet, despite its shortcomings, ABS has a normative hold on the legal understanding of equity.^21^

There are alternatives.

The negotiations for the Pandemic Treaty provide a timely opportunity to reimagine equity and move beyond the path dependency inherent in how equity is conceptualized in the sphere of pandemic preparedness and response. COVID-19 has demonstrated problems with access to pathogenic genetic resources and with access to medical countermeasures such as vaccines, but that does not mean that global health law should connect these issues using ABS. Instead, the Pandemic Treaty negotiations must ensure that all countries have fair and equitable access to medicines — irrespective of whether they can or do contribute pathogens and associated data. At the same time, pathogens, associated data, and the knowledge required to produce medical countermeasures should be shared as openly as feasible. These issues are both crucial, but must be treated as separate, distinct concerns.^22^ Linking them using ABS in the Pandemic Treaty is liable to introduce perverse incentives and adversarial relations, rather than the cooperation and solidarity necessary to achieve more equitable outcomes in future pandemics.

## Conclusion

The negotiators of the Pandemic Treaty have a chance to reconsider and reimagine what equity should look like in a situation where there are fewer vaccine doses than lives at stake. If an ABS system created by the Pandemic Treaty is as vulnerable to nationalistic behavior as COVAX or the PIP Framework, there remains the risk that WHO will not receive medical countermeasures until wealthy countries have taken what they need. The ABS policy mechanism comes cloaked in three decades of rhetoric about equity and fairness, but it is just that — unsupported rhetoric. ABS has proven incapable of delivering equitable outcomes in international environmental law for the last three decades. If Member States genuinely want to achieve anything approaching equitable outcomes in the next pandemic, they must be look to regional capacity building, technology, and know-how transfer before the next pandemic.

## Note

Over the last 36 months, Stephanie Switzer has received funding from the United Nations Convention on Biological Diversity and the Scottish government to undertake studies on areas of law/practice of general relevance to this published piece. The other authors have no conflicts to disclose.

## References

[1] World Health Assembly, “The World Together: Establishment of an Intergovernmental Negotiating Body to Strengthen Pandemic Prevention, Preparedness and Response” 28 November 2021, SSA2/CONF./1Rev.1.

[2] Joint Statement by Heads of States and World Health Organization, “Covid-19 Shows Why United Action Is Needed for More Robust International Health Architecture,” Geneva, Switzerland: World Health Organization, March 30, 2021.

[3] World Health Organization, “Conceptual Zero Draft for the Consideration of the Intergovernmental Negotiating Body at its Third Meeting,” November 25, 2022, A/INB/3/3, Preamble.

[4] World Health Organization, “Zero draft of the WHO CA+ for the consideration of the Intergovernmental Negotiating Body at its fourth meeting,” February 1, 2023, A/INB/4/3, art. 10.

[5] R. Wynberg , “Biopiracy: Crying wolf or a lever for equity and conservation?” Research Policy, 52 no. 104674 (2023): 11.

[6] K. McAfee , “Selling Nature to Save It? Biodiversity and Green Developmentalism,” Environment and Planning D: Society and Space 17, no. 2 (1999).

[7] K. D. Prathapan et al., “When the Cure Kills - CBD Limits Biodiversity Research,” *Scienc*e 360, no. 6396 (2018): 1405–1406. 29954970 10.1126/science.aat9844

[8] World Health Organization, Standard Material Transfer Agreement 2, Annex 2, Article 4.1.1, Pandemic Influenza Preparedness Framework (2011).

[9] World Health Organization, Article 6.9.2, Pandemic Influenza Preparedness Framework (2011).

[10] A. E. Bollinger , “E-MERS-GENCY: An Application and Evaluation of the Pandemic Influenza Preparedness Framework to the Outbreak of MERS-CoV,” Temple International and Comparative Law Journal 29, no. 1 (2015).

[11] D. P. Fidler and L. O. Gostin , “The WHO Pandemic Influenza Preparedness Framework: A Milestone in Global Governance for Health,” Journal of the American Medical Association 306, no. 200 (2011). 10.1001/jama.2011.960 21750298

[12] M. Eccleston-Turner and M. Rourke , “Arguments against the Inequitable Distribution of Vaccines using the Access and Benefit Sharing Transaction,” I*nternational and Comparative Law Quarterly* 70, no. 825 (2021).

[13] B. M. Meier et al., “Travel Restrictions and Variants of Concern: Global Health Laws Need to Reflect Evidence,” Bulletin of the World Health Organization 100, no. 178 (2022). 10.2471/BLT.21.287735 PMC8886257 35261400

[14] O. J. Wouters et al., “Challenges in Ensuring Global Access to COVID-19 Vaccines: Production, Affordability, Allocation, and Deployment,” Lancet 397, no. 1023 (2021). 10.1016/S0140-6736(21)00306-8 PMC7906643 33587887

[15] S. Thambisetty et al., “Addressing Vaccine Inequity during the Covid-19 Pandemic: The TRIPS Intellectual Property Waiver Proposal and Beyond,” Cambridge Law Journal 81, no. 384 (2022).

[16] See Wynberg, *supra* note 5.

[17] World Health Organization, “Report on Influenza Virus Sharing: Report by the Director-General,” 2020, *available at* <https://www.who.int/publications/m/item/WHA72-12-OP1a-Report-Edited_EN> (last visited March 13, 2023).

[18] M. Rourke and M. Eccleston-Turner , “The Pandemic Influenza Preparedness Framework as a ‘Specialized International access and Benefit-Sharing Instrument’ under the Nagoya Protocol,” N*orthern Ireland Legal Quarterly* 72, no. 3 (2021).

[19] World Health Organization, “Meeting of The Pandemic Influenza Preparedness Framework Advisory Group: Report to the Director-General,” October 11-14, 2022.

[20] S. Winands-Kalkuhl and K. Holm-Müller , “Bilateral vs. Multilateral? On the Economics and Politics of a Global Mechanism for Genetic Resource Use,” Journal of Natural Resources Policy Research 7, no. 305 (2015).

[21] S. Laird et al., “Rethink the Expansion of Access and Benefit Sharing: Several UN Policy Processes are Embracing a Calcified Approach to Conservation and Equity in Science,” Science 367, no. 6483 (2020): 1200–1202. 32165574 10.1126/science.aba9609

[22] M. Eccleston-Turner and M. Rourke , “Pathogen Sharing: Balancing Access to Pathogen Samples with Equitable Access to Medicines” in Global Health Law & Policy: Ensuring Justice for a Healthier World (forthcoming 2023).

